# Amyloid-beta induces distinct forms of cell death in different neuronal populations

**DOI:** 10.1038/s41418-025-01649-7

**Published:** 2025-12-15

**Authors:** Rosalind Heron, Clelia Amato, Barbara Monteiro-Black, Robert J. Williams, Will Wood

**Affiliations:** 1 Centre for Inflammation Research, Institute for Regeneration and Repair, 4–5 Little France Drive, The University of Edinburgh, Edinburgh, UK; 2 Department of Life Sciences, University of Bath, Bath, UK

**Keywords:** Cell biology, Neuroscience

## Abstract

Recent FDA approval for treating Alzheimer’s disease (AD) with amyloid-beta (Aβ) immunotherapy is a historic breakthrough, which has rekindled widespread interest in understanding the molecular basis of Aβ toxicity. In this study, we developed a novel *Drosophila* model to investigate Aβ42-induced pathologies in vivo and in real time. Strikingly, we unveiled compelling evidence that secreted Aβ42 affects different neurons in distinct ways—both in susceptibility to Aβ42 deposition and in the mode of cell death triggered. Additionally, we observed altered larval crawling behaviour which—remarkably—could be recovered by inhibiting ferroptotic cell death with small molecule inhibitors. Collectively these findings showcase this as a powerful new model for investigating Aβ toxicity in AD and identifying novel treatment strategies.

## Introduction

A historic breakthrough in Alzheimer’s disease (AD) research and treatment is FDA approval for the use of three passive immunotherapy approaches that target clearance of amyloid-beta (Aβ): aducanumab, lecanemab and, most recently, donanemab [1]. These represent the first disease-modifying interventions approved for the treatment of AD. Though the infamous ‘tau vs. amyloid’ debate over causality persists [2, 3], this decision has refocused widespread demand to understand the early molecular basis of Aβ toxicity in AD [4] and, in doing so, identify additional required therapeutic strategies.

Aβ is a heterogeneous peptide produced through the combined cleavage of the transmembrane amyloid precursor protein (APP) by β-site APP cleaving enzyme (BACE1) and γ-secretase [5, 6]. Aβ release is part of normal cell metabolism [7] and most people show elevated levels of soluble Aβ as they age; however, more accumulated Aβ is observed in AD [8]. Aβ can be cleaved to different lengths, forming isoforms with different numbers of amino acids [9]. Aβ peptides are predominantly cleaved at the C terminus at amino acid 40 to form Aβ40; however, ~10% are cleaved at amino acid 42 which forms Aβ42 [10, 11]. Despite having just two additional amino acids, Aβ42 forms the core component of AD plaques [12, 13] and is associated with AD pathology [14, 15]. Though ultimately accumulating in plaques throughout the brain, increased soluble Aβ42 is an early feature of AD and can even be used for early diagnosis [16, 17]. However, due to the complexity of physiological changes that occur throughout AD progression, it has proved difficult to understand what effects this increase in soluble Aβ42 has on neuronal function. Aβ42 is just one of potentially many features likely to be driving AD pathogenesis. As such, it can be useful to turn to simpler genetic models that allow us to disentangle the Aβ42-mediated pathology in isolation.

In this study, we developed a powerful experimental model that allows the study of Aβ-driven pathology within the fruitfly *Drosophila melanogaster*. We created novel constructs that incorporated a number of genetic sequences designed to optimise functionality and increase concentrations of unmutated human Aβ42 when compared to previous *Drosophila* Aβ models [18–24]. This enabled us to develop an unprecedented *Drosophila* model of neuronal toxicity to Aβ42 that was optimised for tracking the progression of Aβ42-induced pathologies in real time. We use this Aβ model to investigate the deposition of secreted Aβ42 throughout the brain and Aβ42-induced cognitive defects. We characterised immediate Aβ42-induced neuronal death in vivo and demonstrated that this Aβ model can be utilised for identifying new drug treatment strategies. Our results reveal that secreted Aβ42 affects different neuronal subpopulations in distinct ways—both in susceptibility to Aβ42 deposition and in the mode of cell death triggered.

## Materials and methods

### DNA constructs and *Drosophila* stocks

Human Aβ42 (PRO_0000000095: LVFFAEDVGSNKGAIIGLMVGGVVIA) and Aβ40 (PRO_0000000096: LVFFAEDVGSNKGAIIGLMVGGVV) sequences were obtained from UniProt [25] and then codon optimised for *Drosophila* using Java Codon Adaptation Tool (JCat), ensuring that the most common restriction enzymes were removed from the sequences (codon optimised sequences listed in Table S1). In the secretory lines, these sequences were combined with the pre-proenkephalin (PENK) signal sequence as used by Finelli et al. (2004) in their secretory Aβ model [18]. All plasmids were designed identically to the QUAS-PENK::hAβ42 (Secreted hAβ42) plasmid except for the removal of the entire Aβ sequence to create the QUAS-PENK sequence (No Aβ), 2 amino acids at the Aβ C terminus to create the QUAS-PENK::hAβ40 sequence (Secreted hAβ40), or PENK to create the QUAS-hAβ42 sequence (Non-secreted hAβ42). A number of additional sequences were combined to ensure optimal functionality of the plasmids: 15x QUAS to allow for strong expression driven by QF drivers; Kozak2 to allow initiation of translation; with Hsp70 minimal promoter, Mhc intron and SV40 polyA sequences used as described for the refinement of tools for targeted gene expression in *Drosophila* [26]. Another important aspect of plasmid design allowed for a visual readout of the cells that expressed the constructs. For this we incorporated the red fluorophore (mKate2) to all of the PENK/Aβ plasmids. The sequence for mKate2 was obtained from Evrogen and codon optimised for *Drosophila* using JCat (codon optimised sequence listed in Table S1). mKate2 was selected for its reported increased photostability, brightness, quicker folding and lower toxicity than other red fluorescent proteins. The mKate2 fluorophore was not attached to the PENK/Aβ sequences to ensure that it would not affect the function and aggregation of these proteins, however, instead of introducing the mKate2 to these flies separately, we incorporated the fluorophore to the same plasmid to provide a visual way to directly identify the flies and cells that had incorporated and were expressing the PENK/Aβ constructs. The mKate2 sequence was combined with all of the same 15x QUAS, Kozak2, Hsp70, Mhc intron and SV40 polyA sequences as the PENK/Aβ sequences to allow for equally strong expression of this protein when driven by QF drivers.

All of these sequences were assembled in silico, then synthethised by Invitrogen GeneArt Gene Synthesis and cloned into a pw+AttB plasmid using the HindIII and EcoRI restriction enzymes. These novel constructs were then used by BestGene to develop new fly strains.

For labelling of neurons with QUAS-driven constructs, neuronal synaptobrevin QF (nSyb) or embryonic lethal abnormal visual system QF (Elav) were used. The QF fly lines used as part of this work were derived from those ordered through the Bloomington Stock Centre (University of Indiana (NIH P40OD018537)). FlyBase was also used extensively for genetic and molecular information [27]. All *Drosophila* strains were raised at 20 °C on standard cornmeal-agar food at 50–60% relative humidity in a 12:12 h light:dark cycle. All genotypes used in this study are listed in Table S2.

### Survival assay

For synchronisation of embryos, a combination of adult virgins and males of the required phenotypes were put in vials for 1 h with Iberian fly food at 20 °C. The embryos laid during this time were left in the vials to grow and develop. Larvae were checked daily for mortality by assessing response to gentle prod (if no ongoing movement was observed). Upon pupation, pupae were checked daily and the stage of development was recorded. Mortality at this stage of development was determined when the pupae failed to further develop.

### Immunofluorescence

For staining of embryos, synchronised animals were collected by combining adult virgins and males of the required phenotypes at 20 °C in a cage overnight with fresh yeast paste to promote oviposition and using the embryos laid on the resulting apple juice agar plate. Embryos were transfered to cell strainers (Falcon), then dechorionated with bleach before rapidly being fixed in a mixture of heptane (Sigma-Aldrich) and 4% paraformaldehyde (MP Biomedicals) at 1:1 ratio in glass vials. After 30 s of vigorous shaking by hand and a further 30 min on rotating wheel, the fixative was removed and replaced with 100% methanol (Sigma-Aldrich). After vigorously shaking by hand for 30 s, the embryos were transferred in methanol to a 1.5 ml tube (Eppendorf) and washed repeatedly with methanol. The embryos were then washed repeatedly in PBST-2 (PBS containing 0.1% Triton X (Sigma-Aldrich)) solution before overnight incubation at 4 °C with purified mouse anti-β-Amyloid 1-42 monoclonal antibody (BioLegend), diluted 1:200 in PBST-2. The following day, the embryos were washed with PBST-2 and blocked using 2% horse serum (Sigma-Aldrich) diluted in PBST-2 for 30 min on a rotating wheel. After repeated washing with PBST-2, the embryos were incubated for 1 h at room temperature in goat anti-mouse-AlexaFluor^TM^ Plus 488 (Thermo Scientific), diluted 1:200 in PBST-2. After repeated washing in PBST-2, the embryos were transferred to VECTASHIELD mounting medium (Vector Labs) and mounted between a glass slide and a supported coverslip. The slide was then inverted for imaging of the CNS.

For staining of larval and pupal CNS, the brain was dissected from animals and rapidly fixed in cold PBS containing 4% paraformaldehyde (MP Biomedicals) for 30 min. The brains were washed repeatedly with PBST-2 and then transferred to Fish serum blocking buffer (Thermo Scientific) for 30 min. The brains were then washed repeatedly in PBST-2 before overnight incubation at 4 °C with purified mouse anti-β-Amyloid 1-42 monoclonal antibody (BioLegend) diluted 1:200 in PBST-2. The following day, the brains were repeatedly washed with PBST-2 and incubated for 1 h at room temperature in goat anti-mouse-AlexaFluor^TM^ Plus 488 (Thermo Scientific), diluted 1:200 in PBST-2. After repeated washing in PBST-2, the brains were transferred to VECTASHIELD mounting medium (Vector Labs) and mounted between a glass slide and a supported coverslip. The slide was then inverted for imaging.

For imaging fixed samples, a Zeiss LSM 880 confocal microscope was used and a plan-apochromat 40x objective with a NA of 1.3. The acquisition software used was Zen Black (Zeiss). The hard fix preserved the fluorescence of the mKate2-positive neurons, allowing detection in the absence of immunostaining.

### Larval crawling assay and small molecule inhibition

For assessing larval crawling, synchronised animals were collected by combining adult virgins and males of the required phenotypes at 20 °C in a cage overnight with fresh yeast paste to promote oviposition and using the embryos laid on the resulting apple juice agar plate. The plates were supplemented with fresh yeast paste which the larvae had continuous access to for feeding until they were assessed for larval crawling during the second instar stage at 48 h.

At this time, the correct larvae were selected by visualisation of CNS expression of the mKate2 fluorescent protein and carefully transferred to the centre of individual wells in 6-well plates (Corning). Each well was filled two-thirds with 1.5% agarose (Sigma-Aldrich) coloured with Brilliant Black BN (Sigma-Aldrich) to improve contrast against the background. After an acclimation period of ~30 s, larvae were recentred and recording commenced. All larval crawling behaviour was recorded using a Canon EOS M200 camera with an EF-M 15–45 mm IS STM lens mounted in a downward-facing orientation on a fixed tripod. The camera was positioned to fully capture the 6-well plate within the field of view. To minimise glare and reflections, an imaging chamber was used that blocked out all light and noise from the environment. The plate was lit up from the side and placed on a black background to maximise contract during Movie acquisition.

For testing rescue with small molecule inhibitors, the plates of synchronised embryos were supplemented with fresh yeast paste containing either vehicle (0.1% dimethyl sulfoxide (DMSO, Sigma-Aldrich)) or compound (100 μM in 0.1% DMSO). Larvae had continuous access to the yeast paste for feeding until they were assessed for larval crawling behaviour during the second instar stage at 40–48 h. Flavonoids were supplied by Extrasynthese, 2-2-Dipridyl (DPD) was from Sigma-Aldrich, CP502 was a gift from Prof. Robert Hider at King’s College London, and the ferroptosis inhibitors were tested as part of a larger screen of repurposed FDA-approved compounds (Prestwick).

### Live imaging

All live imaging was performed using an inverted spinning disc confocal microscope (PerkinElmer Ultraview) with a Plan-Apochromat 63x objective with a NA of 1.4 and a Hamamatsu C9100-14 camera. The acquisition software used was Volocity (Quorom Technologies). Images of different channels were acquired sequentially, changing the filters between each Z-stack to eliminate bleed through between channels during two- to three-colour imaging.

For embryonic imaging, flies were left at 20 °C in a cage overnight with fresh yeast paste to promote oviposition and the resulting embryos were collected in cell strainers (Falcon). The embryos were then dechorionated with bleach (Jangro) and washed repeatedly with water, before being developmentally staged based on gut morphology (all embryos imaged at stage 16). The embryos were then mounted ventral side up on scotch tape between a glass slide and a supported coverslip in droplets of VOLTALEF oil (VWR Chemicals). The slide was then inverted for imaging of the CNS of the embryo. Z-stacks (20 µm × 0.5–1 µm slices) of the neurons in the CNS were then acquired on the UltraVIEW spinning disc system.

For the microinjection of dyes and inhibitors, embryos were dechorionated, washed and mounted as normal before being dehydrated in a sealed box with silica beads for ~15–30 min at 25 °C. A droplet of VOLTALEF (VWR Chemicals) was added to each embryo before injection into the head of the embryo. Microinjection was performed using an InjectMan4 microinjector (Eppendorf) combined with a FemtoJet 4i injectman rig (Eppendorf) fitted with Femto tips (Eppendorf). A coverslip was sealed on top and imaging undertaken immediately. Annexin V–Alexa Fluor 647 conjugate (Molecular probes, Life Technologies) was injected neat when used alone. For co-labelling with SYTOX^TM^-Green or SYTOX^TM^-Blue (Invitrogen, Thermo Fisher Scientific), a 1/10 dilution of SYTOX^TM^ in phosphate-buffered saline (PBS) (3 µM) was mixed with neat Annexin V-647 at a 1:9 ratio.

### Image analysis

For processing and analysis of larval crawling behaviour, the Movies were first preprocessed in Premiere Pro (Adobe). In the *Effect Controls* panel, the *Mask* feature was used to generate a uniform dark background around the plates. The *Ripple Edit* tool was then used to clip the Movie to 1 min for analysis. The initial portion was removed to eliminate motion artefacts caused by camera adjustment and to ensure that all larvae were actively moving prior to tracking. The trimmed segments were subsequently sped up 40× using the *Time Remapping* tool to reduce file size. The final Movie was exported as an .mp4 file using the *Export Settings* panel (Format: H.264; Frame Size: 1920 × 1080; Frame Rate: 50fps; Duration: 3 s), preserving both image quality and compatibility for downstream analysis in FIJI (National Institute of Health (NIH)). Preprocessed Movies were loaded into FIJI as a virtual stack using the *FFMPEG* importer (File > Import > Movie (FFMPEG)) and all frames included (Frames 0 to −1). To reduce the number of frames and focus on the most informative period of larval movement, the stack was refined using the *Slice Keeper* tool (Image > Stacks > Tools > Slice Keeper). Every second frame within the first minute of the original Movie was retained (Slices 1–47 for imports of 93 frames and Slices 1–62 for imports of 125 frames, both using an Increment of 2), resulting in a final stack of either 23 or 31 frames. A spatial scale was then applied to convert pixel measurements into millimetres. This was achieved by using the *Set Scale* tool (Analyze > Set Scale) to draw a reference line across the diameter of a single well in the 6-well plate, which measures 35 mm (Known Distance: 35; Pixel Aspect Ratio: 1; Unit of Length: mm). Finally, manual tracking was carried out using the *MTrackJ* plugin (Plugins > MTrackJ), from which distance (Len) and velocity (v) measures were obtained. Displacement (Length) was calculated using FIJI’s built-in *Measure* tool (Analyze > Measure), and corresponds to the distance between the first and last points of each track. The total number of head casts and turns were derived by manual counting. Pre-established criteria for exclusion was a displacement of 0 since we had no treatment groups which caused this phenotype, and it was interpreted as an indication of possible injury or stress during transfer to the testing wells.

For processing and analysis of microscopy images, FIJI (NIH) was used. All the analysed and presented microscopy images are maximum intensity z-projections. If required, excessive noise was removed from presented z-projected images using the ‘despeckle’ tool in FIJI, otherwise only the brightness and contrast was adjusted linearly. All analyses were performed on unprocessed images. To quantify fluorescence intensity (Arbitrary Unit, AU) and heterogeneity in the L3 CNS, a 30 × 30 μm^2^ region at the mushroom body of the right hemisphere was divided into 25 equal squares of 6 × 6 μm^2^. Background fluorescence was determined by measuring intensity on a 6 × 6 μm^2^ square outside the CNS from the same image and removed from each square measured in that CNS.

The total number of Annexin V and SYTOX^TM^ labelled soma was derived by manual counting.

### Statistics and reproducibility

The required sample size was calculated by assuming 80% power with significance level of 0.05. All datasets underwent Shapiro-Wilk normality tests to ensure that the appropriate statistical tests were performed. Further comparisons and multiple comparison tests were performed as recommended by the GraphPad Prism software. Area under the survival curves were compared by Kolmogorov-Smirov test. Specific n numbers, statistical tests carried out and their results are reported in Table S3. Post hoc results are shown on graphs and all graphs show mean ± SEM. Prism (GraphPad) was used for all statistical analysis and all figures were configured in Illustrator (Adobe). More information about reagents and resources used throughout the methods can be found in Table S2.

## Results

### Novel *Drosophila* model of aggressive Aβ42 toxicity causes early mortality

**Fig. 1:**
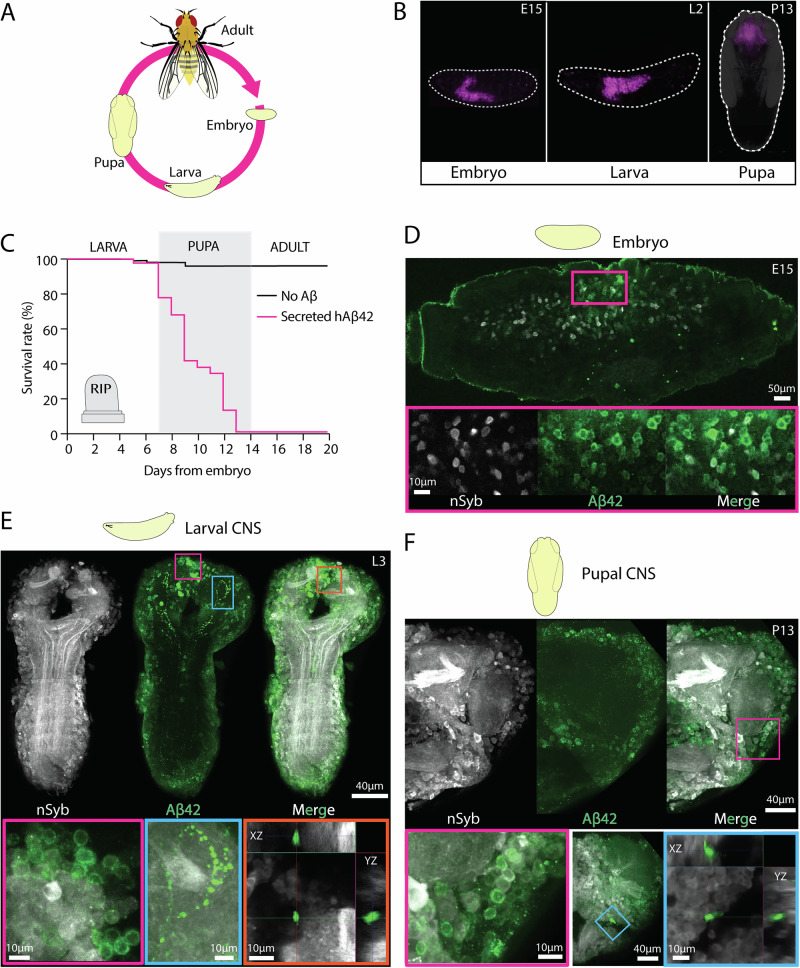
Neurons have varied susceptibility to human Aβ42 deposition in novel *Drosophila melanogaster* model. **A** Diagram showing the life cycle of *Drosophila melanogaster*. Translucency of the *Drosophila* during the three developmental stages of embryo, larva and pupa, make this model ideal for unintrusive live imaging at these stages. **B** Images showing the expression of the mKate2 fluorescent protein being driven by a neuronal nSyb driver. Incorporation of mKate2 into our Aβ plasmid allows us to visualise the neurons that are secreting hAβ42 in the translucent *Drosophila* embryo at stage E15 [Left], larva at stage L2 [Middle] and pupa at stage P13 [Right]. **C** Survival curves of *Drosophila* expressing no Aβ and *Drosophila* secreting hAβ42 neuronally using an nSyb driver. **D** Image of *Drosophila* embryo at stage E15 secreting hAβ42 neuronally [Grey] using an nSyb driver, fixed and stained with Aβ42 primary antibody and Alexa 488 secondary antibody [Green]. Boxed region highlights the varied accumulation of secreted hAβ42 found at the neuronal soma of different neurons in the CNS with inset enlarged in below panels as hAβ42-releasing neurons [Left, Grey], Aβ42 [Middle, Green] and these images overlaid [Right, Grey and Green]. **E** Image showing the dissected CNS from a late third instar *Drosophila* larva (L3) secreting hAβ42 neuronally [Grey] using an nSyb driver, fixed and stained with Aβ42 primary antibody and Alexa 488 secondary antibody [Green]. Pink boxed region highlights continued variation in the accumulated secreted hAβ42 found at the neuronal soma of different neurons in the CNS with inset of Aβ42 staining overlaid with hAβ42-releasing neurons enlarged in pink boxed panel below [Left, Grey and Green]. Blue boxed region highlights punctate-like staining of hAβ42 at certain neuronal axons in the CNS neuropil with inset of Aβ42 staining overlaid with hAβ42-releasing neurons enlarged in blue boxed panel below [Middle, Grey and Green]. Orange boxed region highlights plaque-like staining of hAβ42 found lodged in folds of the brain surface in some third instar larvae with inset enlarged in orange boxed panel below [Right, Green Aβ42 and Grey hAβ42-releasing neurons]. **F** Image showing the right hemisphere of a dissected CNS from *Drosophila* pupa at stage P13 secreting hAβ42 neuronally [Grey] using an nSyb driver, fixed and stained with Aβ42 primary antibody and Alexa 488 secondary antibody [Green]. Pink boxed region highlights continued variation in the accumulated secreted hAβ42 found at the neuronal soma of different neurons in the CNS with inset of Aβ42 staining overlaid with hAβ42-releasing neurons enlarged in pink boxed panel below [Left, Grey and Green]. Lower middle panel shows the same CNS at a different depth where plaque-like staining of Aβ42 is visible lodged in folds at the brain surface, inset enlarged in blue boxed panel beside [Right, Green Aβ42 and Grey hAβ42-releasing neurons].

We developed a novel fly strain designed for investigating Aβ42-induced pathologies in isolation of other hallmarks of AD and permitting us to track the progression of these pathologies in real time using live imaging. We used the neuronal promoter, neuronal Synaptobrevin (nSyb), to drive codon optimised human Aβ42 (hAβ42) fused to the PENK secretory protein—this fusion caused hAβ42 to be immediately released from the neurons. To identify the neurons producing secreted hAβ42, we co-expressed an unattached red fluorescent protein (mKate2) with nSyb. We compared this to a control *Drosophila* strain with no Aβ by using nSyb to drive the same construct with PENK and mKate2, but without hAβ42. Fluorescent microscopy of whole animals allowed us to visualise the mKate2-positive neurons that were driving the PENK/Aβ42 constructs in the CNS of the embryo, larva and pupa (Fig. 1A, B). We found that neuronally-released hAβ42 was highly toxic and caused early mortality in *Drosophila*, before eclosion to adulthood (Fig. 1C). This Aβ42-induced early mortality did not occur at one specific time point, instead, individuals died at different stages between late-stage larvae, and late pupal development. This early mortality illustrates that this novel model of Aβ toxicity has been successfully designed to be more aggressive than previously studied *Drosophila* Aβ models [18–24].

### Neurons have varied susceptibility to Aβ42 deposition

Immunostaining of the CNS with an Aβ42 antibody revealed substantial deposition of secreted hAβ42 at certain neurons, while other neurons displayed no deposition (Fig. 1D–F). This deposition was irrespective of whether or not they were producing hAβ42—indicated by mKate2 expression. Not all of the hAβ42-producing neurons showed deposition of Aβ42. Instead, we observed dense deposition of secreted hAβ42 at select neuronal soma in the embryo, larva and pupa CNS (Fig. 1D–F). There was accompanying punctate-like staining of Aβ42 at distinct neuronal axons in the larval CNS neuropil (Fig. 1E). In some older larvae and pupae, we detected plaque-like staining of Aβ42 lodged in folds at the brain surface (Fig. 1E, F), in possible CNS sanctuary sites. These results reveal that distinct neurons have different susceptibility to the deposition of secreted Aβ42.

### Secreted Aβ42 disrupts larval crawling behaviour

**Fig. 2:**
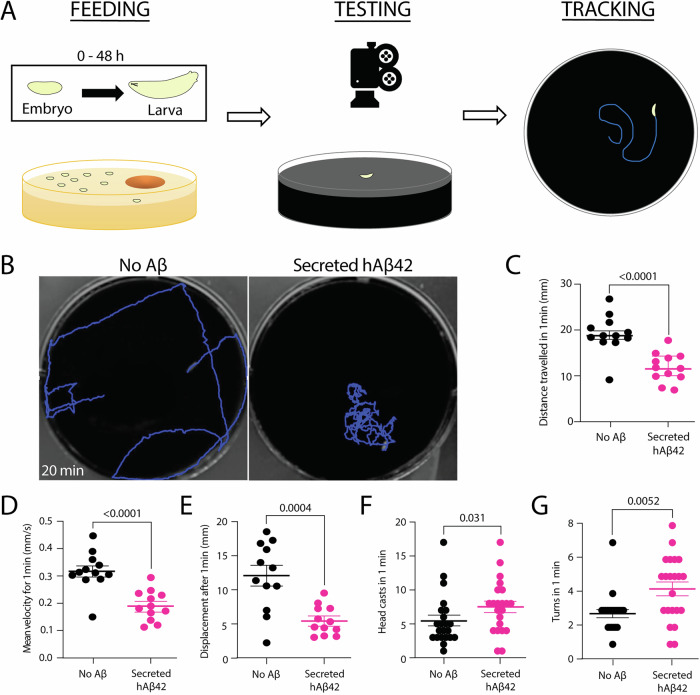
Neuronal secretion of human Aβ42 disrupts larval crawling behaviour in *Drosophila melanogaster.* **A** Diagram outlining the various steps of the larval crawling assay. Left: Embryos are collected on apple juice agar plates by combining adult virgins and males of the required phenotypes in a cage overnight at 20 °C. Fresh yeast paste is added to the plates and the plate is left for 48 h, at which point the embryos have developed into second instar larvae. Middle: At this time, second instar larvae (L2) are carefully transferred into individual wells of a 6-well plate. Each well has a layer of agar at the bottom which is dyed black to improve contrast of images. The behaviour of the larvae is recorded for 1 min and then they are carefully transferred to vials containing Iberian fly food. Right: Movies recording the larval movement during random wandering are used for tracking to detect changes in larval crawling behaviour. **B** Representative tracks for 20 min of larval crawling behaviour in *Drosophila* expressing no Aβ [Left] and *Drosophila* secreting human Aβ42 neuronally using an nSyb driver [Right]. See Supplementary Movie 1 to observe larval crawling behaviour. **C** Distance travelled (mm) during 1 min of second instar larval crawling in *Drosophila* expressing no Aβ and *Drosophila* secreting hAβ42 neuronally using an nSyb driver. **D** Mean velocity (mm/s) for 1 min of second instar larval crawling in *Drosophila* expressing no Aβ and *Drosophila* secreting hAβ42 neuronally using an nSyb driver. **E** Mean displacement (mm) after 1 min of second instar larval crawling in *Drosophila* expressing no Aβ and *Drosophila* secreting hAβ42 neuronally using an nSyb driver. **F** Number of head casts during 1 min of second instar larval crawling in *Drosophila* expressing no Aβ and *Drosophila* secreting hAβ42 neuronally using an nSyb driver. **G** Number of turns during 1 min of second instar larval crawling in *Drosophila* expressing no Aβ and *Drosophila* secreting hAβ42 neuronally using an nSyb driver.

Assessment of larval crawling behaviour (Fig. 2A) highlighted Aβ42-induced reductions in distance travelled, mean velocity and displacement (Fig. 2B–E and Supplementary Movie 1), matching observations noted in previous *Drosophila* Aβ models [18]. Further investigation suggested that the altered crawling behaviour may occur at least in part because neuronally-released hAβ42 disrupts decision-making (Fig. 2F, G and Supplementary Movie 2). *Drosophila* larval decision-making behaviour has been well-categorised, particularly in response to odour [28]. Larval locomotion has two distinct modes: [1] runs, when larvae move forwards in a relatively straight line and [2] turns, when the larva changes direction. Before transitioning between these, a *Drosophila* larva sweeps it’s anterior body from side-to-side in a well-characterised decision-making process known as head casting. The direction of a turn is determined by the position of the final head cast. As the larva moves forward, the rear gradually realigns itself with the front (Supplementary Movie 2). We found Aβ42-induced increases in both larval head casting (Fig. 2F) and turning (Fig. 2G). These results suggest that disrupted decision-making ability may at least partially contribute to the characteristic crawling behaviour observed in Aβ model larvae.

### Toxic effects are specific to the secreted form of Aβ42

**Fig. 3:**
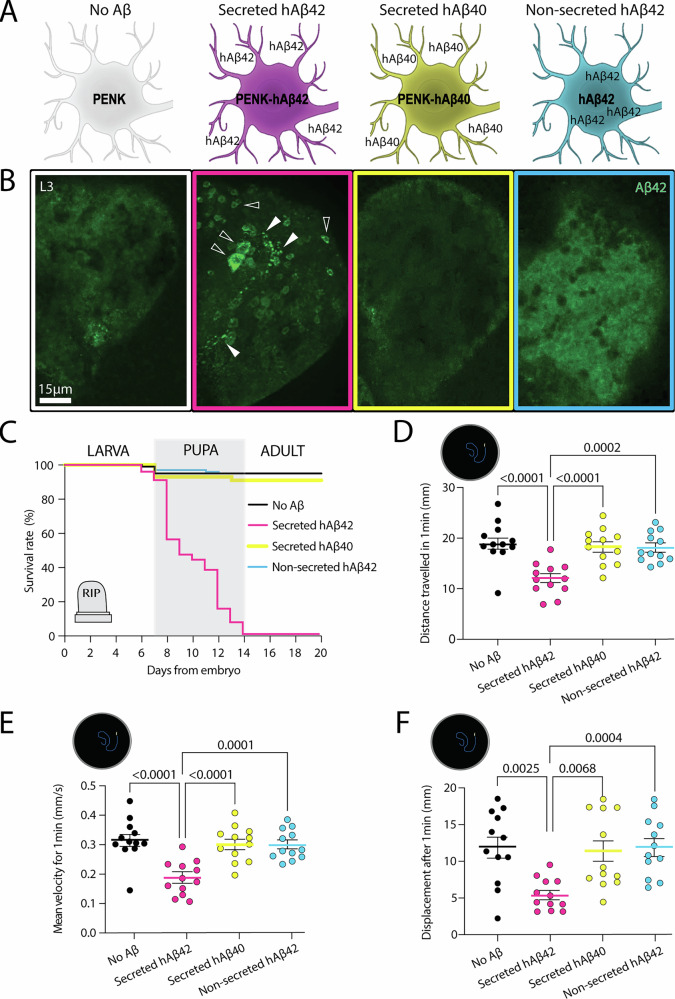
Toxic effects on larval crawling behaviour and mortality in *Drosophila melanogaster* are specific to the secreted Aβ42 isoform. **A** Schematic representing the differences in the Aβ being expressed by neurons using the nSyb driver in our tested strains. In the ‘No Aβ’ strain the PENK secretory peptide is expressed without any attached Aβ, so no Aβ is being expressed or secreted in the neurons [White]. In the ‘Secreted hAβ42’ strain, human Aβ42 is attached to the PENK secretory peptide so it is produced and released from neurons [Pink]. In the ‘Secreted hAβ40’ strain, the shorter, non-toxic human Aβ40 is attached to the PENK secretory peptide so it is produced and released from neurons [Yellow]. In the ‘Non-secreted hAβ42’ strain, human Aβ42 is expressed without the PENK secretory peptide so it is produced but not released from neurons [Blue]. **B** Images showing the right hemisphere from the dissected CNS of later third instar *Drosophila* larvae (L3) fixed and stained with Aβ42 primary antibody and Alexa 488 secondary antibody [Green]. Pink boxed panel highlights localisation of Aβ42 staining at neuronal soma (some indicated with closed white arrows) and punctate-like staining at neuronal axons in the CNS neuropil (some indicated with open white arrows) from larvae secreting hAβ42 neuronally using an nSyb driver. Other panels show that there is no labelling of Aβ42 in the CNS when the nSyb driver is used to drive neuronal expression of no Aβ [White] or secreted hAβ40 [Yellow], and staining appears homogeneously expressed in neurons with non-secreted hAβ42 [Blue]. **C** Survival curves of *Drosophila* expressing nSyb to drive neuronal expression of no Aβ [Black], secreted hAβ42 [Pink], secreted hAβ40 [Yellow] and non-secreted hAβ42 [Blue]. **D** Distance travelled (mm) during 1 min of second instar larval crawling in *Drosophila* expressing nSyb to drive neuronal expression of no Aβ [Black], secreted hAβ42 [Pink], secreted hAβ40 [Yellow] and non-secreted hAβ42 [Blue]. **E** Mean velocity (mm/s) for 1 min of second instar larval crawling in *Drosophila* expressing nSyb to drive neuronal expression of no Aβ [Black], secreted hAβ42 [Pink], secreted hAβ40 [Yellow] and non-secreted hAβ42 [Blue]. **F** Mean displacement (mm) after 1 min of second instar larval crawling in *Drosophila* expressing nSyb to drive neuronal expression of no Aβ [Black], secreted hAβ42 [Pink], secreted hAβ40 [Yellow] and non-secreted hAβ42 [Blue].

Elevated Aβ in the brain is one of the pathological hallmarks of AD [8]. The two main isoforms of Aβ in the brain are the 42-residue Aβ42 and the 40-residue Aβ40 [10, 11]. The hallmark amyloid plaques that accumulate in AD are primarily composed of Aβ42, despite the fact that Aβ40 is more abundant throughout the brain [12, 13]. In fact, Aβ40 is not considered to be toxic despite a mere difference of just two additional hydrophobic amino acids (isoleucine and alanine) at the C-terminus of Aβ42 that are absent in Aβ40 [14, 15]. To demonstrate the specificity of toxic effects on mortality and larval crawling in our Aβ42 model, we used nSyb to drive human Aβ40 (hAβ40) fused to PENK (Fig. 3A). Through immunostaining of larval L3 brains, we confirmed that our Aβ42 antibody was specific to the 42-residue isoform; with significantly lower fluorescence intensity measured in the brains of larvae expressing the 40-residue isoform (Fig. 3B and Fig. S1A). Significantly, we found that neuronal release of the shorter hAβ40 did not cause *Drosophila* to die before eclosion to adulthood (Fig. 3C) and there was no disruption to larval crawling behaviour (Fig. 3D–F and Fig. S1B, C). Our results illustrate that the toxic effects that we observe in our model are specific to the 42-residue Aβ42 isoform, which is known to drive Aβ toxicity in AD.

An unresolved issue in relation to Aβ toxicity is whether or not endosomally-generated Aβ42 that is formed and accumulated (not secreted) inside neurons during AD, contributes directly to disease pathology. Although most Aβ42 is released from neurons following APP cleavage, a distinct pool of non-secreted Aβ42 remains inside neurons of Aβ models and affected individuals [29–31]. Despite evidence that secreted Aβ42 concentrations correlate with disease severity [16, 17], neurodegeneration has also been triggered by blocking autophagy to increase intracellular Aβ42 [32]. To determine if non-secreted neuronal Aβ42 could recapitulate the same toxicity on mortality and larval crawling, we used nSyb to drive human Aβ42 without PENK (Fig. 3A). Immunostaining of the larval L3 brains for Aβ42 did not show the same distinct accumulation of Aβ42 at axons and soma of specific neurons that we observed with the secreted Aβ42 model (Fig. 3B). Instead, Aβ42 staining appears homogeneously expressed in neurons of the CNS in these larvae, although overall mean fluorescence intensity was no different than larval brains from the secreted Aβ42 model (Fig. 3B and Fig. S1A). Interestingly, we found that non-secreted hAβ42 did not cause mortality before eclosion to adulthood (Fig. 3C) or disruption to larval crawling behaviour (Fig. 3D–F and Fig. S1B, C). Our results demonstrate that the toxic effects that we observe in our model are specific to the secreted form of the 42-residue Aβ42 isoform, which is known to drive Aβ toxicity in AD.

### Secreted Aβ42 can induce distinct forms of cell death in different neuronal populations

**Fig. 4:**
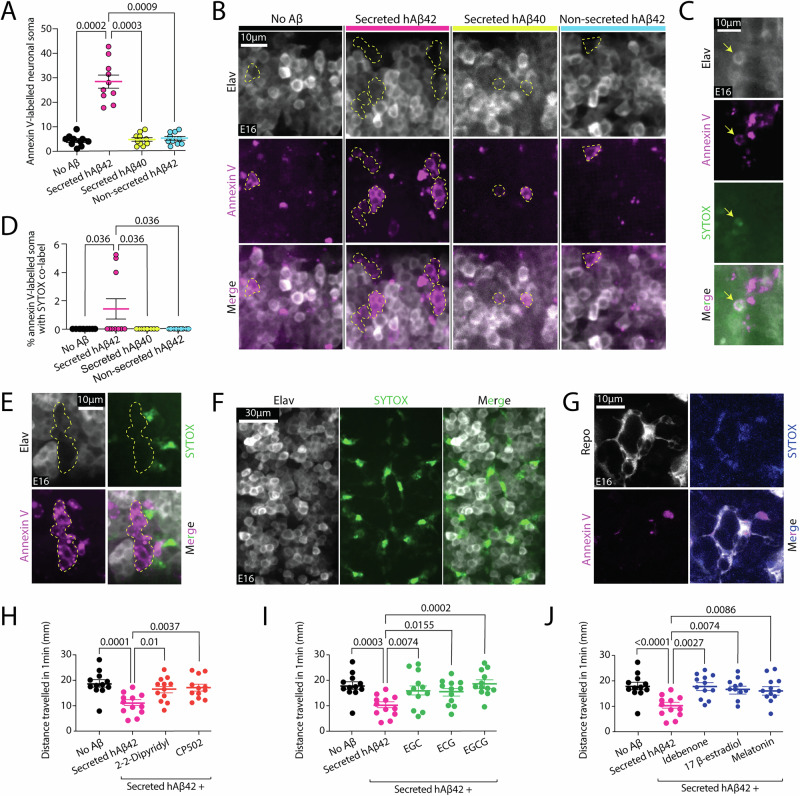
Secreted Aβ42 can induce distinct forms of cell death in different neuronal populations in *Drosophila melanogaster.* **A** Annexin V-labelled death in neuronal soma of *Drosophila* embryos at stage E16 expressing no Aβ [Black], secreted hAβ42 [Pink], secreted hAβ40 [Yellow] and non-secreted hAβ42 [Blue] neuronally using an Elav driver. **B** Live-images of Annexin V-labelled neuronal soma in the CNS of *Drosophila* embryos at stage E16 expressing no Aβ [Black], secreted hAβ42 [Pink], secreted hAβ40 [Yellow] and non-secreted hAβ42 [Blue] neuronally using an Elav driver [Grey] following micro-injection of far-red Annexin V dye [Magenta]. Yellow dashed lines outline regions where there are neuronal gaps in the CNS of neuronal mKate2 fluorescence [Upper, Grey], neurons that have been triggered for Annexin V-associated cell death [Middle, Magenta], and these images overlaid [Lower, Grey and Magenta]. **C** Live-images of a single neuronal soma exhibiting early ferroptotic co-staining in the CNS of *Drosophila* embryos at stage E16 secreting hAβ42 from the neurons using an Elav driver [Upper, Grey] following micro-injection of far-red Annexin V dye [Second, Magenta] and SYTOX^TM^ [Third, Green]. With these images overlaid on the Lower [Grey, Magenta and Green]. Yellow arrow points to a neuronal soma that exhibits early ferroptotic co-labelling. **D** Percentage of Annexin V-labelled neuronal soma undergoing ferroptosis in *Drosophila* embryos at stage E16 expressing no Aβ [Black], secreted hAβ42 [Pink], secreted hAβ40 [Yellow] and non-secreted hAβ42 [Blue] neuronally using an Elav driver. **E** Live-images of Annexin V-labelled neuronal soma in the CNS of *Drosophila* embryos at stage E16 secreting hAβ42 neuronally using an Elav driver [Upper Left, Grey] following co-injection of far-red Annexin V dye [Lower Left, Magenta] and SYTOX^TM^ [Upper Right, Green]. With these images overlaid on the Lower Right [Grey, Magenta and Green]. Yellow dashed lines outline regions where there are gaps in the CNS of neuronal mKate2 fluorescence. **F** Live-images of SYTOX^TM^ labelling in the CNS of *Drosophila* embryos at stage E16 secreting hAβ42 neuronally using an Elav driver [Left, Grey] following injection of SYTOX^TM^ [Middle, Green]. These images are overlaid on the Right [Grey and Green]. **G** Live images of SYTOX^TM^ and Annexin V labelling localised in the glia [Upper Left, Grey] of *Drosophila* embryos at stage E16 following micro-injection of far-red Annexin V dye [Lower Left, Magenta] and SYTOX^TM^ [Upper Right, Blue]. These images are overlaid on the Lower Right [Grey, Magenta and Blue]. **H** Distance travelled (mm) during 1 min of second instar larval crawling in *Drosophila* expressing no Aβ and *Drosophila* secreting hAβ42 neuronally using an nSyb driver in larvae fed yeast paste with vehicle or larvae fed yeast paste containing iron chelators. **I** Distance travelled (mm) during 1 min of second instar larval crawling in *Drosophila* expressing no Aβ and *Drosophila* secreting hAβ42 neuronally using an nSyb driver in larvae fed yeast paste with vehicle or larvae fed yeast paste containing flavonoids. **J** Distance travelled (mm) during 1 min of second instar larval crawling in *Drosophila* expressing no Aβ and *Drosophila* secreting hAβ42 neuronally using an nSyb driver in larvae fed yeast paste with vehicle or larvae fed yeast paste containing repurposed FDA-approved compounds with reported anti-ferroptosis properties.

We have previously established live imaging of the *Drosophila* embryo as a powerful system for shedding new light on the molecular mechanisms underlying tissue damage [33]. Here, we utilised these techniques to identify immediate Aβ42-induced neuronal loss. Using the neuronal driver, Elav (embryonic lethal abnormal visual system), to co-express secreted hAβ42 and mKate2 earlier in development enabled prolonged live imaging of the developing embryo. Microinjection of far-red Annexin V into embryos, combined with live imaging, revealed immediate increases in Aβ42-induced neuronal death in the brain (Fig. 4A, B). Annexin V, is a commonly used marker of apoptotic corpses due to its high affinity for phosphatidylserine (PS), which translocates to the outer plasma membrane during early apoptosis [34]. Annexin V-labelled neuronal death was observed—mostly in individual neurons or in pairs—in control, secreted Aβ40 and non-secreted Aβ42 embryos at levels representative of expected apoptotic neuronal death during normal developmental remodelling [35] (Fig. 4A, B). However, we observed striking increases in Annexin V-labelled neuronal death in immediate response to neuronally-released hAβ42. Interestingly, most of this increased Aβ42-induced neuronal death occurred in groups of greater than 2 neurons (Fig. 4B). These groups of Annexin V-labelled neurons had also lost the mKate2 fluorescence in all embryos tested (Fig. 4B), revealing that these neurons were losing their fluorescence when triggered to undergo apoptotic cell death. This suggests that the Aβ42-positive/mKate2-negative neurons observed in Fig. 1D–F were likely undergoing apoptosis.

We also observed a small number of Aβ42-induced Annexin V-positive single neurons which were mKate2-positive. We hypothesised that these were undergoing a different form of cell death. Having previously demonstrated that co-labelling with SYTOX^TM^ and Annexin V is a reliable marker for ferroptosis in both *Drosophila* and mammalian cells [36], we utilised this technique for further investigation. Co-injecting green SYTOX^TM^ with far-red Annexin V, revealed a small number of single neurons co-labelled for both of these markers, confirming that these neurons were indeed undergoing ferroptosis (Fig. 4C, D). Notably, there was no SYTOX^TM^ labelling visible in Aβ42-induced groups of Annexin V-labelled neurons, as previously described (Fig. 4E). Interestingly we observed high levels of SYTOX^TM^ labelling throughout the brain that did not co-localise with neurons (Fig. 4F). We predicted that this labelling corresponded to glial cells that engulf dead and dying cells within the CNS as part of normal development. We confirmed this through injection of blue SYTOX^TM^ alongside far-red Annexin V into flies expressing gfp within the glia using a Repo driver. Subsequent imaging clearly revealed both Annexin V and SYTOX^TM^ labelled debris within the Repo-positive glial cells (Fig. 4G). Altogether, these results reveal that secreted Aβ42 triggers distinct modes of cell death in different neurons.

### Inhibition of ferroptotic cell death rescues larval crawling behaviour

Given our results demonstrating the presence of Aβ42-induced ferroptosis, and its recent emergence as an important player in AD pathology [37–39], we targeted this mode of cell death with a number of small molecule inhibitors to see if we could rescue larval crawling behaviour. We fed larvae compounds with previously reported anti-ferroptosis properties [40–44] before assessing larval crawling behaviour. Remarkably, compounds targeting different stages of the ferroptotic pathway could all, individually, rescue larval crawling behaviour (Fig. 4H–J and Fig. S2A–F). Feeding Aβ42 model larvae with synthesised metal chelating agents, 2-2-Dipyridyl (DPD) and CP502, designed to reduce cellular iron levels by binding with the iron to form a complex that is less easily dissociated [45–48], completely recovered larval crawling behaviour (Fig. 4H and Fig. S2A, D). The same effect was observed upon feeding larvae with flavonoids—a group of naturally occurring polyphenols with both antioxidant and iron chelator properties [49, 50] (Fig. 4I and Fig. S2B, E). These compounds have previously been shown to reverse cognitive dysfunction and suppress the progression of AD pathology in rodent models [51–54]. Specifically, the flavanols, epigallocatechin (EGC), epicatechin gallate (ECG) and epigallocatechin gallate (EGCG), all rescued larval crawling behaviour. Lastly, feeding larvae repurposed FDA-approved drugs that have previously reported anti-ferroptosis properties [40–44], idebenone, 17-β-estradiol and melatonin, all rescued hAβ42-induced larval crawling behaviour (Fig. 4J and Fig. S2C, F). Our results establish this novel Aβ model as a powerful tool for discovering new drug treatment strategies and shows that Aβ42-induced ferroptosis in a subset of neurons in vivo is sufficient to drive rapid cognitive decline in *Drosophila*.

## Discussion

Elevated Aβ42 has been considered a hallmark of AD since the discovery that the characteristic plaques in an AD brain primarily consisted of Aβ42 [55, 56]. However, due to the complexity of this disease and difficulty of capturing cellular changes in real time, it has proved difficult to tease apart the Aβ42-evoked toxicity from other pathologies. The recent FDA approval for using Aβ immunotherapies to treat AD has refocused widespread interest in understanding the molecular basis of Aβ toxicity [4].

We developed a novel *Drosophila* model optimised for investigating Aβ42-induced pathologies in isolation of other hallmarks of AD and tracking their progression in real time. We revealed that secreted Aβ42 was deposited at specific neurons irrespective of whether or not those neurons were producing it. In addition, we found that not all Aβ42-producing neurons exhibited this deposition of secreted Aβ42. This substantiates that different neurons have varied susceptibility to secreted Aβ42. This difference in neuronal susceptibility to Aβ42 was also evident when we investigated immediate Aβ42-induced neuronal death in vivo. We unveiled immediate increases in Aβ42-induced neuronal death—the majority of which appeared to be apoptotic, with select neurons undergoing ferroptosis. We also demonstrated the potential of our novel Aβ model for future drug treatment strategies, by using small molecule inhibitors of cell death to rescue Aβ42-disrupted larval crawling behaviour.

The cognitive symptoms of AD result from damage to the neurons and synapses involved in regulating memory and cognition—with substantial brain atrophy evident in affected individuals due to increased neuronal loss [57–59]. Previous studies have shown that elevated Aβ can cause neuronal loss and synaptic dysfunction in vitro and in transgenic animal models [60–66]. Here, we confirmed that elevated Aβ42 could induce neuronal death, and revealed that this can occur at a rapid rate. It is not yet clear how Aβ42 causes neuronal death, with evidence suggesting it could be due to a range of factors, such as, disrupting neuronal communication by interfering with synaptic function [67], forming pores in neuronal membranes [68], or as a result of triggering abnormal tau aggregation [69]. Thanks to the ease of genetic manipulation and live imaging, this novel *Drosophila* Aβ model provides an ideal platform to investigate how Aβ42 induces neuronal death in vivo.

Since recent scientific progress has ignited a new appreciation of the diversity of cell death and led to standardised classification of different modes of regulated cell death (RCD) [70], multiple modes of RCD have been implicated in AD [71, 72]. Here, we demonstrate that elevated Aβ42 can activate distinct modes of cell death in different neurons at the same time. Most of the cell death at this early timepoint appears to be apoptotic, with a small number of neurons undergoing ferroptosis. Ferroptosis has recently emerged as an important player in AD pathology, with some ferroptosis inhibitors already showing therapeutic promise for AD in pre-clinical models [37–39]. Of course, due to the wide-acting nature of different compounds that can be classified as inhibitors of ferroptosis, there could be additional indirect factors contributing to their therapeutic effect. Notably, these compounds could also provide rescue of symptoms through their global antioxidant activity or by reducing other forms of cell death, such as apoptosis through altered metabolism or signalling pathways. As such, these could be factors that indirectly contribute to the rescued Aβ42-induced larval crawling observed in our Aβ model. Nevertheless, by testing compounds that targeted different stages of the ferroptosis pathway in different ways, and finding that they could all rescue altered Aβ42-induced larval crawling behaviour, we conclude that inhibition of ferroptosis plays a central role in this rescue. Here, we demonstrate for the first time that neuronal Aβ42 secretion alone is sufficient to drive ferroptosis in vivo, and that inhibiting Aβ42-induced ferroptosis can rescue cognitive defects.

Our work demonstrates that there is much to be understood about how a neuron’s identity contributes to its mode of cell death, an aspect that has previously been difficult to delve into. This novel *Drosophila* model of Aβ42 toxicity could prove to be a useful tool for determining the differences between neurons that are not susceptible to Aβ42 and neurons that are. A recent whole organism snRNA-seq *Drosophila* study highlighted that sensory neurons of the head, such as auditory sensory neurons and olfactory receptor neurons, are particularly vulnerable to Aβ42 [73]. These could be factors contributing to the changes in larval crawling behaviour observed in both this and *Drosophila* models targeting earlier points of the Aβ-producing pathway (Fig. 2B–E and Supplementary Movie 1, [74–76]). Though our observation of increased head casting and turning behaviour suggests that neurons involved in decision-making may also be vulnerable to Aβ42 (Fig. 2F, G and Supplementary Movie 2) and worth further exploring.

In conclusion, our results reveal that secreted Aβ42 affects different neurons in distinct ways—both in susceptibility to Aβ42 deposition and in the mode of cell death triggered. Further, our results exhibit that this novel Aβ model is a powerful tool for discovering new drug treatment strategies and show that Aβ42-induced ferroptosis in a subset of neurons in vivo is sufficient to drive rapid cognitive decline in *Drosophila*. This work provides exciting new insight into the cellular response to Aβ toxicity and paves the way for future study into how neuronal identity influences how that cell dies. Collectively these findings showcase our system as a powerful new model for investigating Aβ toxicity in AD and for potentially identifying future drug treatment strategies.

## Supplementary information

Supplemental Figure legends

Figure S1

Figure S2

Table S1

Table S2

Table S3

Movie 1

Movie 2

## Data Availability

All relevant data is presented in the main manuscript or additional supporting files. Newly developed flies will be made available to the fly community upon request to the corresponding author.
