# Supplementary material for: Amyloid-beta induces distinct forms of cell death in different neuronal populations
Source: Cell Death Differ. 2025 Dec 15;33(7):1345–55. doi: 10.1038/s41418-025-01649-7 (PMC13341879; doi:10.1038/s41418-025-01649-7)
Supplement: Supplementary file 1 — Supplemental Figure legends [file 41418_2025_1649_MOESM1_ESM.docx]

**Supplemental figure legends**

**Figure S1. Toxic effects on larval head casting and turning in *Drosophila melanogaster* are specific to the secreted Aβ42 isoform**

A. Fluorescence intensity of CNS stained with Aβ42-specific antibody from third instar larvae of *Drosophila* expressing nSyb to drive neuronal expression of no Aβ [Black], secreted hAβ42 [Pink], secreted hAβ40 [Yellow] and non-secreted hAβ42 [Blue].

B. Number of head casts during 1 min of second instar larval crawling in *Drosophila* expressing nSyb to drive neuronal expression of no Aβ [Black], secreted hAβ42 [Pink], secreted hAβ40 [Yellow] and non-secreted hAβ42 [Blue].

C. Number of turns during 1 min of second instar larval crawling in *Drosophila* expressing nSyb to drive neuronal expression of no Aβ [Black], secreted hAβ42 [Pink], secreted hAβ40 [Yellow] and non-secreted hAβ42 [Blue].

**Figure S2. Toxic effects on larval head casting and turning in *Drosophila melanogaster* can be rescued by ferroptosis inhibitors**

A. Number of head casts during 1 min of second instar larval crawling in *Drosophila* expressing no Aβ and *Drosophila* secreting hAβ42 neuronally using an nSyb driver in larvae fed yeast paste with vehicle or larvae fed yeast paste containing iron chelators.

B. Number of head casts during 1 min of second instar larval crawling in *Drosophila* expressing no Aβ and *Drosophila* secreting hAβ42 neuronally using an nSyb driver in larvae fed yeast paste with vehicle or larvae fed yeast paste containing flavonoids.

C. Number of head casts during 1 min of second instar larval crawling in *Drosophila* expressing no Aβ and *Drosophila* secreting hAβ42 neuronally using an nSyb driver in larvae fed yeast paste with vehicle or larvae fed yeast paste containing repurposed FDA-approved compounds with reported anti-ferroptosis properties.

D. Number of turns during 1 min of second instar larval crawling in *Drosophila* expressing no Aβ and *Drosophila* secreting hAβ42 neuronally using an nSyb driver in larvae fed yeast paste with vehicle or larvae fed yeast paste containing iron chelators.

E. Number of turns during 1 min of second instar larval crawling in *Drosophila* expressing no Aβ and *Drosophila* secreting hAβ42 neuronally using an nSyb driver in larvae fed yeast paste with vehicle or larvae fed yeast paste containing flavonoids.

F. Number of turns during 1 min of second instar larval crawling in *Drosophila* expressing no Aβ and *Drosophila* secreting hAβ42 neuronally using an nSyb driver in larvae fed yeast paste with vehicle or larvae fed yeast paste containing repurposed FDA-approved compounds with reported anti-ferroptosis properties.

**Supplemental Table 1. Codon optimised sequences**

**Supplemental Table 2. List of all reagents and resources**

**Supplemental Table 3. Statistics and general methods**

**Supplementary Movie 1. Disrupted larval crawling behaviour in *Drosophila melanogaster* secreting human Aβ42 neuronally**

Movie showing 20 min of second instar larval crawling behaviour of *Drosophila* expressing no Aβ [Left, White] and *Drosophila* secreting hAβ42 neuronally [Right, Pink] using an nSyb driver. Movies are played at 40x speed.

**Supplementary Movie 2. Observing head casts and turns associated with decision-making in *Drosophila melanogaster* in control and Aβ42 model larvae**

Movie showing crawling behaviour of *Drosophila* second instar larvae expressing no Aβ [Left, White] and *Drosophila* secreting hAβ42 neuronally [Right, Pink] using an nSyb driver. Movies are played in real time.
