# Supplementary figures and images for: Amyloid-beta induces distinct forms of cell death in different neuronal populations
Source: Cell Death Differ. 2025 Dec 15;33(7):1345–55. doi: 10.1038/s41418-025-01649-7 (PMC13341879; doi:10.1038/s41418-025-01649-7)

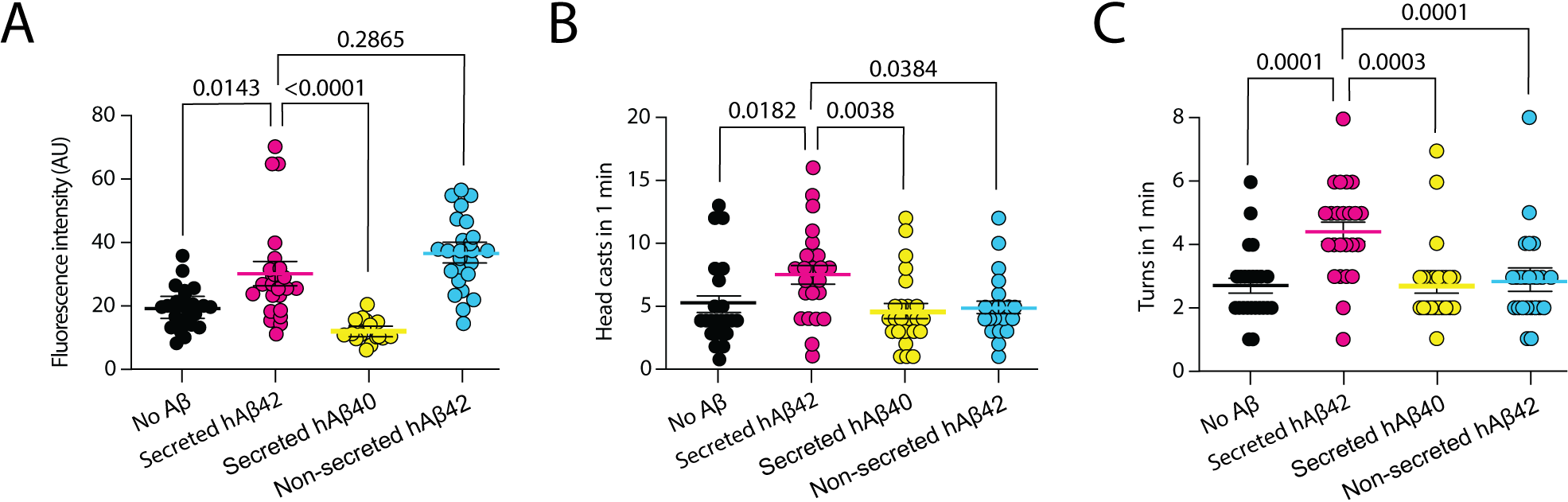

Supplement: Supplementary file 2 — Figure S1 [file 41418_2025_1649_MOESM2_ESM.png]

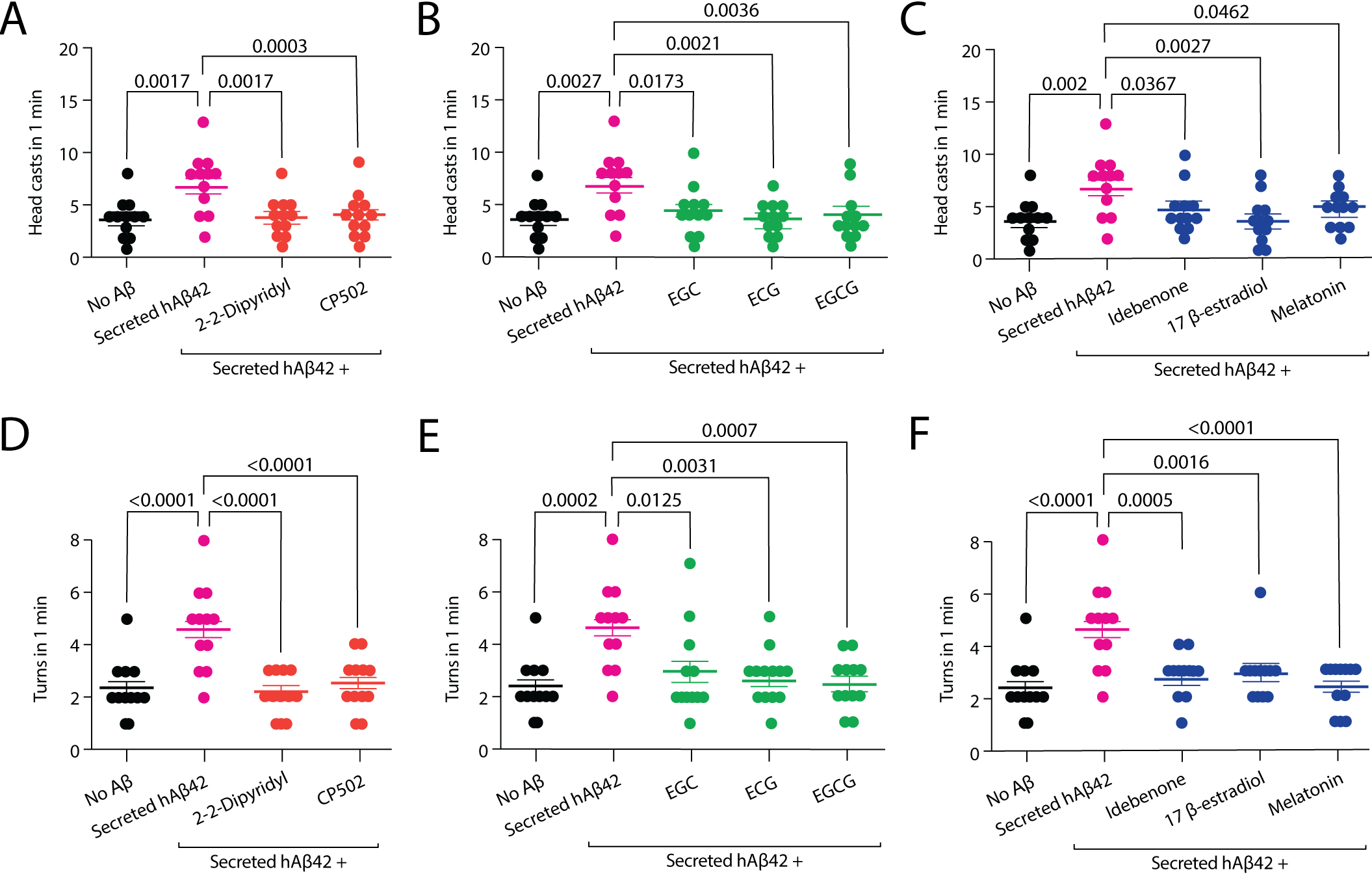

Supplement: Supplementary file 3 — Figure S2 [file 41418_2025_1649_MOESM3_ESM.png]
