# Supplementary material for: Amyloid-beta induces distinct forms of cell death in different neuronal populations
Source: Cell Death Differ. 2025 Dec 15;33(7):1345–55. doi: 10.1038/s41418-025-01649-7 (PMC13341879; doi:10.1038/s41418-025-01649-7)
Supplement: Supplementary file 4 — Table S1 [file 41418_2025_1649_MOESM4_ESM.docx]

Supplemental Table 1: Codon optimised sequences

| **Protein** | **Codon optimised sequence** |
| --- | --- |
| Human Aβ42 | GGCGATCACCACGCCGCCCACCATCAGGCCGATGATGGCGCCCTTGTTGGAGCCCACGTCCTCGGC GAAGAACACCAGCTTCTGGTGGTGCACCTCGTAGCCGGAGTCGTGGCGGAACTCGGCGTC |
| Human Aβ40 | CACCACGCCGCCCACCATCAGGCCGATGATGGCGCCCTTGTTGGAGCCCACGTCCTCGGCGAAGAA CACCAGCTTCTGGTGGTGCACCTCGTAGCCGGAGTCGTGGCGGAACTCGGCGTC |
| mKate2 | GCGGTGGCCCAGCTTGGAGGGCAGGTCGCAGTAGCGGGCCACGGCCACCTCGTGCTGCTCCACGT  AGGTCTCCTTGTCGGCCTCCTTGATGCGCTCCAGGCGGCGGTCCACGTAGTACACGCCGGGCATCTT  CAGGTTCTTGGCGGGCTTCTTGGAGCGGTAGGTGGTCTTCAGGTTGCAGATCAGGTGGCCGCCGCC  CACCAGCTTCAGGGCCATGTCGGCGCGGCCCTCCAGGCCGCCGTCGGCGGGGTACAGGGTCTCGG  TGGAGGCCTCCCAGCCCAGGGTCTTCTTCTGCATCACGGGGCCGTTGGAGGGGAAGTTCACGCCGC  GGATCTTCACGTTGTAGATCAGGCAGCCGTCCTGCAGGGAGGTGTCCTGGGTGGCGGTCAGCACG CCGCCGTCCTCGTAGGTGGTCACGCGCTCCCAGGTGAAGCCCTCGGGGAAGGACTGCTTGAAGAA  GTCGGGGATGCCCTGGGTGTGGTTGATGAAGGTCTTGGAGCCGTACATGAAGGAGGTGGCCAGGA TGTCGAAGGCGAAGGGCAGGGGGCCGCCCTCCACGGCCTTGATGCGCATGGTCTGGGTGCCCTCG TAGGGCTTGCCCTCGCCCTCGGAGGTGCACTTGAAGTGGTGGTTGTTCACGGTGCCCTCCATGTACA GCTTCATGTGCATGTTCTCCTTGATCAGCTCGGACACCAT |
