# Supplementary material for: Amyloid-beta induces distinct forms of cell death in different neuronal populations
Source: Cell Death Differ. 2025 Dec 15;33(7):1345–55. doi: 10.1038/s41418-025-01649-7 (PMC13341879; doi:10.1038/s41418-025-01649-7)
Supplement: Supplementary file 5 — Table S2 [file 41418_2025_1649_MOESM5_ESM.docx]

| Supplemental Table 2. List of all reagents and resources | |  |
| --- | --- | --- |
|  |  |  |
| **REAGENT or RESOURCE** | **SOURCE** | **IDENTIFIER** |
| **Antibodies and probes** | | |
| Purified mouse anti-b-Amyloid 1-42 monoclonal antibody | BioLegend | 805501 |
| Goat anti-Mouse IgG (H+L) Highly Cross-Adsorbed Secondary Antibody, Alexa Fluor™ Plus 488 | Thermo Scientific | A32723TR |
| Annexin V, Alexa Fluor 647 conjugate | Molecular probes, Life Technologies | A23204 |
| SYTOX™ Green Nucleic Acid Stain - 5 mM Solution in DMSO | Invitrogen, Thermo Fisher Scientific | S7020 |
| SYTOX™ Blue Nucleic Acid Stain - 5 mM Solution in DMSO | Invitrogen, Thermo Fisher Scientific | S11348 |
| **Chemicals** | | |
| Heptane | Sigma-Aldrich | 34873 |
| Paraformaldehyde, 16% aqueous solution, methanol free | MP Biomedicals | 30525-89-4 |
| Methanol | Sigma-Aldrich | 34860 |
| 0.1% Triton X | Sigma-Aldrich | T8787 |
| 2% horse serum | Sigma-Aldrich | H0146 |
| VECTASHIELD antifade mounting medium for fluorescence | Vector Labs | H-1000-10 |
| Fish serum blocking buffer | Thermo Scientific | 37527 |
| Agarose | Sigma-Aldrich | A9539 |
| Brilliant Black BN | Sigma-Aldrich | 211842 |
| Dimethyl sulfoxide (DMSO) | Sigma-Aldrich | 20-139 |
| Bleach | Jangro | CK945 |
| Oil 10 S, VOLTALEF | VWR Chemicals | 24627.188 |
| **Experimental models: Organisms/strains** | | |
| *D. melanogaster*: nSyb-QF: y[1] w[1118];; M{w[+mC]=nSyb-QF2.P}ZH-86Fb/TM6B,Tb[1]; | Bloomington Drosophila Stock Center | RRID: BDSC_51955; FlyBase: FBti0154977 |
| *D. melanogaster*: Elav-QF: P{w[+mW.hs] RFP[mCh.3xP3.cPa ]=ET-QF2.GB}elav[C155-QF2]; betaTub60D[Pin-1]/CyO;; | Bloomington Drosophila Stock Center | RRID: BDSC_66466; FlyBase: FBst0066466 |
| *D. melanogaster*: repo-Gal4: w[1118];P{w[+m*]=GAL4}repo  /TM3, Sb[1]; | Bloomington Drosophila Stock Center | RRID: BDSC_7415; FlyBase: FBst0007415 |
| *D. melanogaster*: No Aβ: ;QUAS-PENK,QUAS-mKate2;; | Novel strain |  |
| *D. melanogaster*: Secreted hAβ42: ;QUAS-PENK-hAβ42,QUAS-mKate2;; | Novel strain |  |
| *D. melanogaster*: Secreted hAβ40: ;QUAS-PENK-hAβ40,QUAS-mKate2;; | Novel strain |  |
| *D. melanogaster*: Non-secreted hAβ42: ;QUAS-hAβ42,QUAS-mKate2;; | Novel strain |  |
| *D. melanogaster*: UAS-GFP ;UAS-GFP::NES-T2A-H2B::Venus[VK27][M2];; | Novel strain |  |
| **Compounds** | | |
| 2,2'-Dipridyl | Sigma-Aldrich | D216305 |
| CP502 | Gift from Hider Lab, KCL |  |
| (-)-Epigallocatechin | Extrasynthese | 0979 S |
| (-)-Epigallocatechin gallate | Extrasynthese | 0981 S |
| (-)-Epicatechin gallate | Extrasynthese | 0978 S |
| Idebenone | Prestwick | Prestw-1288 |
| 17 β-estradiol | Prestwick | Prestw-441 |
| Melatonin | Prestwick | Prestw-458 |
| **Software** | | |
| Zen Black | Zeiss | https://www.zeiss.com/microscopy/en/products/software/zeiss-zen.html |
| Volocity | Quorom | www.volocity4d.com |
| Premiere Pro | Adobe | www.adobe.com/uk/products/premiere.html |
| ImageJ2/FIJI V2.14.0/1.54f | National Institute of Health | imagej.net/ij/ |
| GraphPad Prism V10.4.0 | GraphPad Software | www.graphpad.com/scientific-software/prism/ |
| Illustrator | Adobe | www.adobe.com/uk/products/illustrator.html |
| UniProt | Uniprot | www.uniprot.org |
| Java Codon Adaptation Tool | Prodoric | www.jcat.de |
