# Supplementary material for: Amyloid-beta induces distinct forms of cell death in different neuronal populations
Source: Cell Death Differ. 2025 Dec 15;33(7):1345–55. doi: 10.1038/s41418-025-01649-7 (PMC13341879; doi:10.1038/s41418-025-01649-7)
Supplement: Supplementary file 6 — Table S3 [file 41418_2025_1649_MOESM6_ESM.docx]

| Supplemental Table 3: Statistics and general methods | | |
| --- | --- | --- |
|  |  |  |
| **Figure panels** | **Sample size and collection (n)** | **Statistical methods and measures** |
| 1C | nSyb-QF,QUAS-PENK,QUAS-mKate2: n=200 animals;  nSyb-QF,QUAS-PENK-Aβ42,QUAS-mKate2: n=200 animals | Kolmogorov-Smirnov test, two-sided, p=0.0002 |
| 2C | nSyb-QF,QUAS-PENK,QUAS-mKate2: n=12 larvae;  nSyb-QF,QUAS-PENK-Aβ42,QUAS-mKate2: n=12 larvae | Unpaired t-test, two-sided, p<0.0001 |
| 2D | nSyb-QF,QUAS-PENK,QUAS-mKate2: n=12 larvae;  nSyb-QF,QUAS-PENK-Aβ42,QUAS-mKate2: n=12 larvae | Unpaired t-test, two-sided, p<0.0001 |
| 2E | nSyb-QF,QUAS-PENK,QUAS-mKate2: n=12 larvae;  nSyb-QF,QUAS-PENK-Aβ42,QUAS-mKate2: n=12 larvae | Unpaired t-test with Welch's correction, two-sided, p=0.0009 |
| 2F | nSyb-QF,QUAS-PENK,QUAS-mKate2: n=22 larvae;  nSyb-QF,QUAS-PENK-Aβ42,QUAS-mKate2: n=22 larvae | Mann-Whitney U Test, two-sided, p=0.031 |
| 2G | nSyb-QF,QUAS-PENK,QUAS-mKate2: n=22 larvae;  nSyb-QF,QUAS-PENK-Aβ42,QUAS-mKate2: n=22 larvae | Unpaired t-test with Welch's correction, two-sided, p=0.0052 |
| 3C | nSyb-QF,QUAS-PENK,QUAS-mKate2: n=200 animals;  nSyb-QF,QUAS-PENK-Aβ42,QUAS-mKate2: n=200 animals;  nSyb-QF,QUAS-PENK-Aβ40,QUAS-mKate2: n=200 animals;  nSyb-QF,QUAS-Aβ42,QUAS-mKate2: n=200 animals | Kolmogorov-Smirnov test, two-sided, p=0.0002 |
| 3D | nSyb-QF,QUAS-PENK,QUAS-mKate2: n=12 larvae;  nSyb-QF,QUAS-PENK-Aβ42,QUAS-mKate2: n=12 larvae;  nSyb-QF,QUAS-PENK-Aβ40,QUAS-mKate2: n=12 larvae;  nSyb-QF,QUAS-Aβ42,QUAS-mKate2: n=12 larvae | One-way ANOVA with Dunnett’s MC test, p<0.0001 |
| 3E | nSyb-QF, QUAS-PENK,QUAS-mKate2: n=12 larvae;  nSyb-QF,QUAS-PENK-Aβ42, QUAS-mKate2: n=12 larvae;  nSyb-QF,QUAS-PENK-Aβ40,QUAS-mKate2: n=12 larvae;  nSyb-QF,QUAS-Aβ42,QUAS-mKate2: n=12 larvae | One-way ANOVA with Dunnett’s MC test, p<0.0001 |
| 3F | nSyb-QF,QUAS-PENK,QUAS-mKate2: n=12 larvae;  nSyb-QF,QUAS-PENK-Aβ42,QUAS-mKate2: n=12 larvae;  nSyb-QF,QUAS-PENK-Aβ40,QUAS-mKate2: n=12 larvae;  nSyb-QF,QUAS-Aβ42,QUAS-mKate2: n=12 larvae | Welch's ANOVA with Dunnett’s MC test, p<0.0001 |
| 4A | nSyb-QF,QUAS-PENK,QUAS-mKate2: n=10 embryos;  nSyb-QF,QUAS-PENK-Aβ42,QUAS-mKate2: n=10 embryos;  nSyb-QF,QUAS-PENK-Aβ40,QUAS-mKate2: n=10 embryos;  nSyb-QF,QUAS-Aβ42,QUAS-mKate2: n=10 embryos | Kruskal-Wallis with Dunn's MC test, p<0.0001 |
| 4E | nSyb-QF,QUAS-PENK,QUAS-mKate2: n=10 embryos;  nSyb-QF,QUAS-PENK-Aβ42,QUAS-mKate2: n=10 embryos;  nSyb-QF,QUAS-PENK-Aβ40,QUAS-mKate2: n=10 embryos;  nSyb-QF,QUAS-Aβ42,QUAS-mKate2: n=10 embryos | Kruskal-Wallis with Dunn's MC test, p=0.0237 |
| 4H | nSyb-QF,QUAS-PENK,QUAS-mKate2 + vehicle: n=12 larvae;  nSyb-QF,QUAS-PENK-Aβ42,QUAS-mKate2 + vehicle: n=12 larvae;  nSyb-QF,QUAS-PENK-Aβ42,QUAS-mKate2 + 2-2-Dipyridyl: n=12 larvae;  nSyb-QF,QUAS-PENK-Aβ42,QUAS-mKate2 + CP502: n=12 larvae | One-way ANOVA with Dunnett’s MC test, p=0.0004 |
| 4I | nSyb-QF,QUAS-PENK,QUAS-mKate2 + vehicle: n=12 larvae;  nSyb-QF,QUAS-PENK-Aβ42,QUAS-mKate2 + vehicle: n=12 larvae;  nSyb-QF,QUAS-PENK-Aβ42,QUAS-mKate2 + Epigallocatechin (EGC): n=12 larvae;  nSyb-QF,QUAS-PENK-Aβ42,QUAS-mKate2 + Epicatechin gallate (ECG): n=12 larvae;  nSyb-QF,QUAS-PENK-Aβ42,QUAS-mKate2 + Epigallocatechin gallate (EGCG): n=12 larvae | One-way ANOVA with Dunnett’s MC test, p=0.0003 |
| 4J | nSyb-QF,QUAS-PENK,QUAS-mKate2 + vehicle: n=12 larvae;  nSyb-QF,QUAS-PENK-Aβ42,QUAS-mKate2 + vehicle: n=12 larvae;  nSyb-QF,QUAS-PENK-Aβ42,QUAS-mKate2 + Idebenone: n=12 larvae;  nSyb-QF,QUAS-PENK-Aβ42,QUAS-mKate2 + 17 β-estradiol: n=12 larvae;  nSyb-QF,QUAS-PENK-Aβ42,QUAS-mKate2 + Melatonin: n=12 larvae | One-way ANOVA with Dunnett’s MC test, p=0.0004 |
| S1A | nSyb-QF,QUAS-PENK,QUAS-mKate2: n=25 ROIs;  nSyb-QF,QUAS-PENK-Aβ42,QUAS-mKate2: n=25 ROIs;  nSyb-QF,QUAS-PENK-Aβ40,QUAS-mKate2: n=25 ROIs;  nSyb-QF,QUAS-Aβ42,QUAS-mKate2: n=25 ROIs | Kruskal-Wallis with Dunn's MC test, p<0.0001 |
| S1B | nSyb-QF,QUAS-PENK,QUAS-mKate2: n=24 larvae;  nSyb-QF,QUAS-PENK-Aβ42,QUAS-mKate2: n=24 larvae;  nSyb-QF,QUAS-PENK-Aβ40,QUAS-mKate2: n=24 larvae;  nSyb-QF,QUAS-Aβ42,QUAS-mKate2: n=24 larvae | Kruskal-Wallis with Dunn's MC test, p=0.0059 |
| S1C | nSyb-QF,QUAS-PENK,QUAS-mKate2: n=24 larvae;  nSyb-QF,QUAS-PENK-Aβ42,QUAS-mKate2: n=24 larvae;  nSyb-QF,QUAS-PENK-Aβ40,QUAS-mKate2: n=24 larvae;  nSyb-QF,QUAS-Aβ42,QUAS-mKate2: n=24 larvae | Kruskal-Wallis with Dunn's MC test, p<0.0001 |
| S2A | nSyb-QF,QUAS-PENK,QUAS-mKate2 + vehicle: n=12 larvae;  nSyb-QF,QUAS-PENK-Aβ42,QUAS-mKate2 + vehicle: n=12 larvae;  nSyb-QF,QUAS-PENK-Aβ42,QUAS-mKate2 + 2-2-Dipyridyl: n=12 larvae;  nSyb-QF,QUAS-PENK-Aβ42,QUAS-mKate2 + CP502: n=12 larvae. | One-way ANOVA with Dunnett’s MC test, p=0.0009 |
| S2B | nSyb-QF,QUAS-PENK,QUAS-mKate2 + vehicle: n=12 larvae;  nSyb-QF,QUAS-PENK-Aβ42,QUAS-mKate2 + vehicle: n=12 larvae;  nSyb-QF,QUAS-PENK-Aβ42,QUAS-mKate2 + Epigallocatechin (EGC): n=12 larvae;  nSyb-QF,QUAS-PENK-Aβ42,QUAS-mKate2 + Epicatechin gallate (ECG): n=12 larvae;  nSyb-QF,QUAS-PENK-Aβ42,QUAS-mKate2 + Epigallocatechin gallate (EGCG): n=12 larvae | One-way ANOVA with Dunnett’s MC test, p=0.0021 |
| S2C | nSyb-QF,QUAS-PENK,QUAS-mKate2 + vehicle: n=12 larvae;  nSyb-QF,QUAS-PENK-Aβ42,QUAS-mKate2 + vehicle: n=12 larvae;  nSyb-QF,QUAS-PENK-Aβ42,QUAS-mKate2 + Idebenone: n=12 larvae;  nSyb-QF,QUAS-PENK-Aβ42,QUAS-mKate2 + 17 β-estradiol: n=12 larvae;  nSyb-QF,QUAS-PENK-Aβ42,QUAS-mKate2 + Melatonin: n=12 larvae | One-way ANOVA with Dunnett’s MC test, p=0.0036 |
| S2D | nSyb-QF,QUAS-PENK,QUAS-mKate2 + vehicle: n=12 larvae;  nSyb-QF,QUAS-PENK-Aβ42,QUAS-mKate2 + vehicle: n=12 larvae;  nSyb-QF,QUAS-PENK-Aβ42,QUAS-mKate2 + 2-2-Dipyridyl: n=12 larvae;  nSyb-QF,QUAS-PENK-Aβ42,QUAS-mKate2 + CP502: n=12 larvae. | One-way ANOVA with Dunnett’s MC test, p=0.0004 |
| S2E | nSyb-QF,QUAS-PENK,QUAS-mKate2 + vehicle: n=12 larvae;  nSyb-QF,QUAS-PENK-Aβ42,QUAS-mKate2 + vehicle: n=12 larvae;  nSyb-QF,QUAS-PENK-Aβ42,QUAS-mKate2 + Epigallocatechin (EGC): n=12 larvae;  nSyb-QF,QUAS-PENK-Aβ42,QUAS-mKate2 + Epicatechin gallate (ECG): n=12 larvae;  nSyb-QF,QUAS-PENK-Aβ42,QUAS-mKate2 + Epigallocatechin gallate (EGCG): n=12 larvae | One-way ANOVA with Dunnett’s MC test, p=0.0004 |
| S2F | nSyb-QF,QUAS-PENK,QUAS-mKate2 + vehicle: n=12 larvae;  nSyb-QF,QUAS-PENK-Aβ42,QUAS-mKate2 + vehicle: n=12 larvae;  nSyb-QF,QUAS-PENK-Aβ42,QUAS-mKate2 + Idebenone: n=12 larvae;  nSyb-QF,QUAS-PENK-Aβ42,QUAS-mKate2 + 17 β-estradiol: n=12 larvae;  nSyb-QF,QUAS-PENK-Aβ42,QUAS-mKate2 + Melatonin: n=12 larvae | One-way ANOVA with Dunnett’s MC test, p<0.0001 |
| All graphs represent 1 replicate | | |
